# Pianeta Nutrizione kids: international pediatric conference on food, physical activity, growth and well-being: Milan, Italy. 25-27 June 2015

**DOI:** 10.1186/s13052-016-0240-0

**Published:** 2016-06-06

**Authors:** Massimo Agosti, Carlo Agostoni, Serge Chalons, Pascale Chavatte-Palmer, José Manuel Moreno Villares, Sophie Nicklaus, Luís Pereira-da-Silva, Angelo Pietrobelli, the MeNu Group, Marie Françoise Rolland-Cachera, Gian Vincenzo Zuccotti, Marco Cappa, Manuela Caruso-Nicoletti, Elena Inzaghi, Stefano Cianfarani, Mario De Curtis, Laura Guazzarotti, Lorenzo Iughetti, Francesco Chiarelli, Laura Comegna, Simone Franchini, Laura Perrone, Giuseppina Rosaria Umano, Elisabetta Petrella, Raffaele Bruno, Valentina Bertarini, Giulia Pedrielli, Isabella Neri, Fabio Facchinetti, Flavia Prodam, Alessandro Sartorio, John M. H. Buckler, Nicoletta Marazzi, Maria E. Street, Viviana D. Patianna, Paola Accorsi, Sara Lo Scocco, Sergio Amarri, Alberto Pellai, Giampiero Merati, Isabella Merzagora, Susanna Rampichini, Arsenio Veicsteinas, Mario Clerici, Paolo Manzoni, Elena Tavella, Elena Boano, Daniele Farina, Fabio Pace

**Affiliations:** Reparto di Nido, Neonatologia e Terapia Intensiva Neonatale, Pediatria del Verbano, Azienda ospedaliera, Polo universitario, Varese, Italy; Department of Clinical Sciences and Community Health, University of Milan, IRCCS Ospedale Maggiore Policlinico, Milano, Italy; Fort de France, France; Maisons Alfort, Paris, France; INRA (National Institute of Agronomical Research), Paris, France; University of Queensland, Paris, France; Hospital 12 de Octubre, Universidad Complutense, Madrid, Spain; Center for Taste and Feeding Behaviour (Centre des Sciences du Goût et de l’Alimentation, CSGA), INRA (French National Institute of Agronomical Research), Dijon, France; NOVA Medical School, Universidade Nova de Lisboa, Lisbon, Portugal; NICU, Hospital Dona Estefânia, Centro Hospitalar de Lisboa Central, Lisbon, Portugal; Pediatric Unit, Verona University Medical School, Verona, Italy; Pennington Biomedical Research Center, Baton Rouge, LA USA; Paris 13 University, Equipe de Recherche en Epidémiologie Nutritionnelle, Centre de Recherche en Epidémiologie et Statistiques, Inserm (U1153), Inra (U1125), Cnam, COMUE Sorbonne Paris Cité, F-93017 Bobigny, France; Clinica Pediatrica, Ospedale dei Bambini V. Buzzi, Milano, Italy; Department University Hospital, Unit of Endocrinology an Diabetes, Bambino Gesù Children’s Hospital, IRCCS, Rome, Italy; Dipartimento di Medicina Clinica e Sperimentale, Sezione di Pediatria, AOU Policlinico-Vittorio Emanuele, Catania, Italy; Dipartimento Pediatrico Universitario Ospedaliero Bambino Gesù Children’s Hospital, Università di Roma Tor Vergata, Rome, Italy; Department of Women’s and Children’s Health, Karolinska Institutet, Stockholm, Sweden; Department of Pediatrics and Pediatric Neuropsychiatry, La Sapienza University of Rome, Rome, Italy; Department of Pediatric, Luigi Sacco Hospital, University of Milan, Milan, Italy; Postgraduate School of Pediatrics, University of Modena and Reggio Emilia, Modena, Italy; Department of Pediatrics, University of Chieti, Chieti, Italy; Clinical Research Centre, University of Chieti, Chieti, Italy; Department of Women and Children and General and Specialized Surgery, Seconda Università degli Studi di Napoli, Naples, 80138 Italy; Mother-Infant Department, University of Modena and Reggio Emilia, Modena, Italy; Division of Pediatrics, Department of Health Sciences, Università del Piemonte Orientale, Novara, 28100 Italy; Endocrinology, Department of Translational Medicine, Università del Piemonte Orientale, Novara, 28100 Italy; Istituto Auxologico Italiano, Milan, Italy; Istituto Auxologico Italiano, Verbania, Italy; Department of Paediatrics, IRCCS- Arcispedale S. Maria Nuova, Reggio Emilia, Italy; Medical Sciences Department, University of Milano, Milano, Italy; Department of Biomedical Sciences for Health, Università degli Studi di Milano, Milan, Italy; Sport Medicine Center, Fondazione Don Gnocchi, Milan, Italy; Medicina Legale, Dipartimento di Scienze Biomediche per la Salute, University of Milan, Milan, Italy; Department of Biomedical Sciences for Health, University of Milan, Milan, Italy; Department of Physiology, University of Milan, Milan, Italy; School in Molecular and Translational Medicine, University of Milano, Milano, Italy; Department of Molecular medicine and Imaging for Rehabilitation, IRCCS SM Nascente, Fondazione Don C Gnocchi, Milano, Italy; Neonatology and NICU, S. Anna Hospital, Torino, Italy; UOC Gastroenterologia Ospedale “Bolognini”, Seriate, BG Italy

## Abstract

A1 Preterm and low birth weight nutrition in the first month life: implications for the outcome

Massimo Agosti

A2 Behind human milk and breastfeeding: not only food, not only growth

Carlo Agostoni

A3 To prevent obesity: importance and issues of cultural adaptation from weaning to 3 years of age

Serge Chalons

A4 Diet before and during pregnancy and child health: lessons from animal models

Pascale Chavatte-Palmer

A5 Infant nutrition: an opportunity to optimize future health

José Manuel Moreno Villares

A6 Complementary feeding strategies to facilitate acceptance of fruits and vegetables

Sophie Nicklaus

A7 Diet of young children in the Mediterranean region

Luís Pereira-da-Silva

A8 Proposal of 10 good practices to help prevent obesity in the first 1,000 days

Angelo Pietrobelli, the MeNu Group

A9 Macronutrient intakes in early life and subsequent risk of obesity

Marie Françoise Rolland-Cachera

A10 The burden of childhood obesity in Italy and the results of Nutrintake study

Gian Vincenzo Zuccotti

A11 Growth body composition and growth hormone therapy: linear growth

Marco Cappa

A12 Early nutrition pattern and late metabolic consequences

Manuela Caruso-Nicoletti

A13 Nutrition and Insulin-like Growth Factor (IGF) System

Elena Inzaghi, Stefano Cianfarani

A14 Nutrition of preterm infants

Mario De Curtis

A15 Early nutrition patterns and later metabolic outcomes- I part: Genetic and metabolic mechanisms

Laura Guazzarotti

A16 Diagnosis of metabolic disease by imaging techniques

Lorenzo Iughetti

A17 Nutrition, growth and cardiovascular diseases

Francesco Chiarelli, Laura Comegna, Simone Franchini

A18 Body fat mass and gender

Laura Perrone, Giuseppina Rosaria Umano

A19 Lifestyle interventions for an appropriate birth weight

Elisabetta Petrella, Raffaele Bruno, Valentina Bertarini, Giulia Pedrielli, Isabella Neri, Fabio Facchinetti

A20 Nutrition, growth and body composition

Flavia Prodam

A21 Nation-specific reference growth charts in the daily practice

Alessandro Sartorio, John M. H. Buckler, Nicoletta Marazzi

A22 Growth patterns in inflammatory bowel diseases (IBD) and in cystic fibrosis (CF)

Maria E. Street, Viviana D. Patianna, Paola Accorsi, Sara Lo Scocco, Sergio Amarri

A23 Newborn in the digital era and their body feeling: physical exercise to counteract hyperphagia

Alberto Pellai

A24 Nutrition, young athletes and effects of exercise. Practical suggestions

Giampiero Merati

A25 Physical exercise as a way to prevent criminality in minors and teenagers

Isabella Merzagora

A26 The measurement of daily energy expenditure in children. Evaluation of a new wrist portable device vs breath-by-breath metabolimeter

Susanna Rampichini, Arsenio Veicsteinas

A27 Probiotic and inflammasomes

Mario Clerici

A28 Probiotics and newborns

Paolo Manzoni, Elena Tavella, Elena Boano, Daniele Farina

A29 Relationship between gut microbiota and obesity

Fabio Pace

## 1000 days

### A1 Preterm and low birth weight nutrition in the first month life: implications for the outcome

#### Massimo Agosti

##### Reparto di Nido, Neonatologia e Terapia Intensiva Neonatale, Pediatria del Verbano, Azienda ospedaliera, Polo universitario, Varese, Italy

Preterm babies need of quantity and quality of nutrients to permit a growth rate, in terms of weight and of body composition to that of a fetus of the same gestational age. Protein and energy administration is the driver for growth. Preterms accumulate a deficit in energy and protein and extrauterine growth retardation begins. Studies support the role of early nutritional support in the first days of life, relating both to the neurological development and to auxological growth later on. Nutrients need to be administered early after delivery and parenteral nutrition should be started and combined with a small amount of trophic feeding to quicken gut maturantion. Catch-up growth occurs when an individual, after a period of growth restriction, tries to return towards his original growth channel. The actual potential for catch-up growth is not well known. Preterms have a different body composition at term corrected age compared with infants born at term, with a significant relative deficit in lean tissue but a fat mass distribution similar to term infants. All preterms should receive human milk because it has a wide range of beneficial effects; fortifiers provide energy which enables preterms to meet their energy needs while keeping feeding volumes low. If breast milk is not available, preterm formulas are suggested in order to supply more protein and energy, besides calcium.

### A2 Behind human milk and breastfeeding: not only food, not only growth

#### Carlo Agostoni (carlo.agostoni@unimi.it)

##### Department of Clinical Sciences and Community Health, University of Milan, IRCCS Ospedale Maggiore Policlinico, Milano, Italy

The effects of feeding human milk are still discussed, since they seem to overcome what can be expected from its chemical composition. On the other hand also the attribution of the main effects to breastfeeding as behavior cannot explain effects such as protection from infections and some autoimmune disorders.

Observational studies for over 50 years have shown associations between early breastfeeding and improved measures of neurodevelopmental outcome [1]. The question of nature *vs* nurture in determining the developmental effects of the early type of feeding remains open since trials are possible only in particular conditions, such as prematurity and non-availability of own’s mother milk. On the whole, available data point to a beneficial effect of human milk on neurodevelopment. While docosahexaenoic acid, naturally represented in human milk, has been given a role in this bioprocess, iron in human milk is low, even if well absorbed, and cannot account for the differences in neurocognitive achievement between breastfed and formula fed infants.

The control of early growth (or, better, weight) acceleration processes is considered today a first measure to control the early appearance of overweight and obesity. Human milk seems to have protective effects, and low basal insulin levels are found in 9 months old infants in an inverse relationship with the number of daily breastfeeding episodes [2]. Recently, the higher protein intakes observed in formula fed infants have been given a role in increasing the synthesis of growth-promoting factors.

Human milk, beyond its mere chemical composition, seems to have a compositional balance able to match both aspects in a favourable manner. Besides these biochemical and physiological effects, also the interactivity promoted by breastfeeding between the infant and the mother and caregivers may have a relevant additional role.

Infants who are fed from the breast can control milk intake, because they are the ones who decide when to start and when to stop sucking. Mothers who breastfeed might develop a feeding style that is less controlling, thereby allowing their infants to maintain their natural ability to regulate their energy intake. This behavior has been found inversely related to breastfeeding duration [3]. Infants who are bottle-fed are less likely on the other hand to control their milk intake. Nevertheless, the virtuous circle of the roles of nature vs nurture in the health effects of breastfeeding cannot be easily disentangled.

**References**

[1] Anderson JW, Johnstone BM, Remley DT: **Breast-feeding and cognitive development: a meta-analysis**. *Am J Clin Nutr* 1999, **70**:525–35.

[2] Madsen AL, Larnkjar A, Molgaard C, Michaelsen KF: **IGF-I and IGFBP-3 in healthy 9 month old infants from the SKOT cohort: breastfeeding, diet, and later obesity**. *Growth Horm IGF Res* 2011, **21**:199–204.

[3] Taveras EM, Scanlon KS, Birch L, Rifas-Shiman SL, Rich-Edwards JW, Gillman MW: **Association of breastfeeding with maternal control of infant feeding at age 1 year**. *Pediatrics* 2004, **114**:577–83.

### A3 To prevent obesity: importance and issues of cultural adaptation from weaning to 3 years of age

#### Serge Chalons (schalons88@gmail.com)

##### Fort de France, France

Early prevention of obesity in children is of major importance because once installed its reduction is often problematic, contributing to the global epidemic growing steadily. The prevention of obesity in children is closely linked to the quality of food in the early years. Besides dietary aspect that queries the type, quality and quantity of food ingested by the child, or physical activity within reach, there is the cultural component that seems dominant, as a risk factor obesity. Around the world, the epidemic of obesity is growing by the advance of a global culture strongly influenced by Western subculture of urban areas, with special features both, food content and how to eat. The weaning period is particularly interesting since the child will integrate food codes that will influence the entire development. In this case these codes favor sweet, hyper protein, salty, ready to eat, leaving the fruits and vegetables, all in a context that has changed the rituals around food.

### A4 Diet before and during pregnancy and child health: lessons from animal models

#### Pascale Chavatte-Palmer

##### Maisons Alfort, France, INRA (National Institute of Agronomical Research), France, University of Queensland, France

Epidemiological studies have shown that early nutrition plays a seminal role in determining offspring’s long-term health and metabolism. The study of children born to women having suffered the Dutch famine during WW2 has clearly highlighted the role of maternal nutrition during pregnancy. Current concerns focus on the increased incidence of obesity and diabetes prior and during pregnancy in humans. Animal models give the possibility to pinpoint critical periods of development, assess the role of specific nutrients and decipher mechanisms. Although rodents are essential for fundamental biological explorations, other species can offer more physiological resemblance to humans. Using a rabbit model, we showed that maternal high fat diet administered to females from before puberty affects the onset of puberty and follicular reserve in the ovary. Although fertility is not affected, early embryos accumulate lipid droplets within the trophoblast, which is accompanied by disturbed gene expression. During gestation, sex-specific perturbations of fetal and placental metabolism are observed. Offspring develop components of the metabolic syndrome as adults with alterations in ovarian follicular reserve as well as testicular function. These data and those of others demonstrate the importance of maternal nutrition not during gestation, in the periconceptional period and before conception.

### A5 Infant nutrition: an opportunity to optimize future health

#### José Manuel Moreno Villares

##### Hospital 12 de Octubre, Universidad Complutense, Madrid, Spain

Since almost 30 years it has been proposed that disease risk and body functions are programmed during critical early periods of development. Humans have sensitive windows for nutrition in terms of later health outcomes. Dietary factors in pregnant and lactating women and during infancy can modulate growth and functional development and exert life-long programming effects on health or disease risk.

Recent interventional studies have tested this nutritional programming hypothesis and new paradigms in nutrition during pregnancy and early life have been set according to these findings. The immediate postnatal life is a vulnerable time for permanent metabolic programming.

Breastfeeding, time and characteristics of complementary feeding, learning of feeding skills in the first year of life have been shown to be related to future health outcomes: dismissed risk of infectious diseases during the first years of life, a decrease in the risk of autoimmune diseases, better nutritional habits, etc. On the contrary, an excess in protein intake in the first 2 years of life is associated with higher prevalence of overweight in childhood and later on, or decreased levels of vitamin D a higher risk of atopy and asthma as an example.

Although there is a long way to know exactly which are the biological mechanisms underlying these findings, there are practical messages to take home: if babies received an appropriate feeding within the first two years of life -and even before, during fetal period- where variety and moderation are combined, as well as physical activity, it will be most likely to have healthier long-term lives.

### A6 Complementary feeding strategies to facilitate acceptance of fruits and vegetables

#### Sophie Nicklaus

##### Center for Taste and Feeding Behaviour (Centre des Sciences du Goût et de l’Alimentation, CSGA), INRA (French National Institute of Agronomical Research), Dijon, France

Complementary feeding (CF), which should begin after exclusive breastfeeding for 6 months, according to the WHO, or after 4 months and before 6 months according to the ESPGHAN, is a period when the infant learns implicitly what, when and how to eat and how much of a given food to eat. At the onset of CF, the brain and the gut are still developing and maturing, and food experiences contribute to shape brain connections involved in food hedonics and in the control of food intake. These learning processes are likely to have a long-term impact. Children’s consumption of fruits and vegetables are below recommendations in many countries. Thus, it is crucial to establish preferences for these foods early, when infant are learning to eat. The development of food preferences mainly starts when infants discover their first solid foods. CF accustoms an infant to the foods in his family’s diet, which will ultimately constitute the basis of his own diet. The factors that influence fruit and vegetable acceptance at the start of the CF period will be described: previous breastfeeding experience; timing of introduction to complementary foods; repeated exposures to the food; variety of foods offered as of the start of the complementary feeding period; quality and sensory properties of the complementary foods; quality of the meal time context; parental responsive feeding.

### A7 Diet of young children in the Mediterranean region

#### Luís Pereira-da-Silva (l.pereira.silva@netcabo.pt)^1,2^

##### ^1^NOVA Medical School, Universidade Nova de Lisboa, Portugal; ^2^NICU, Hospital Dona Estefânia, Centro Hospitalar de Lisboa Central, Lisbon, Portugal

Despite a common Mediterranean dietary pattern, diversity in culture, ethnic background, religion, economy and agricultural production has resulted in different diets among at least 16 countries around the Mediterranean basin. Agricultural policies in European Union in particular have encouraged the production of sugar, fats, oils, and meat at low cost through subsidies and other measures, compared with the limited market supply of fruit and vegetables.

This overview is centered on recent published data on the dietary intake of young children living in European Union Mediterranean countries. Representative results from the multinational European IDEFICS study as well as national studies such as the EPACI and studies from Geração XXI cohort in Portugal, ALSALMA in Spain, EDEN cohort in France, Nutrintake-636 in Italy and GENESIS in Greece, are addressed.

In many European Mediterranean countries young children consume a reasonable or even satisfactory amount of fruit and vegetables, despite high prevalence of overweight/obesity in most cases. This may be related to simultaneous high energy intake, including extra energy intake from sugared beverages and snacks as well as high than recommended protein intake. Excessive sodium intake is found in many countries, being an additional concern. Low adherence to Mediterranean diet by preschool children is currently found in European Mediterranean countries, which in turn is associated to overweight/obesity. Unhealthier diets of young children are associated with lower maternal educational level and unemployment status. Early consumption of energy-dense foods and overweight seem to track across toddler and preschool ages.

Programs attempting to improve adherence to the traditional Mediterranean diet by young children should be part of multi-intervention strategy for prevention and treatment of overweight/obesity. Breastfeeding should be encouraged, since longer breastfeeding is related to higher fruit and vegetable intake in future.

The impact of the current economic crises in European Union Mediterranean countries on young children’s diet and nutritional status is a matter of concern and needs monitoring.

### A8 Proposal of 10 good practices to help prevent obesity in the first 1,000 days

#### Angelo Pietrobelli^1,2^, the MeNu Group

##### ^1^Pediatric Unit, Verona University Medical School, Verona, Italy; ^2^Pennington Biomedical Research Center, Baton Rouge, LA, USA

The prevalence of childhood overweight and obesity has increased in the majority of countries in the last decades. Thinking in a simplistic way, we can consider that obesity would be the result of an imbalance between energy intake and energy expenditure. Also, environment from conception to childhood could influence the child’s future health. The first 1,000 days of life start with woman’s pregnancy and offer an unique window of opportunity to contribute to obesity prevention.

Aim of this presentation is to discuss a proposal of 10 good practices to help prevent obesity in the first 1,000 days. **1**. Both mother and father behavior matter. A balanced diet with adapted excessive fat and protein intake and favoring fruits and vegetables is recommended for both parents during conception period and pregnancy. Furthermore, overweight/obese women who plan to be pregnant should reduce weight before conception. **2**. Before and during pregnancy, at birth and during early life, body composition measurements are crucial to monitor growth. **3**. The exclusive breastfeeding is recommended at the beginning of life until six months. **4**. Four to six months of age is the optimal window to introduce complementary feeding. Until one year of age, breast milk or follow-on formula must remain the main source of feeding and cow’s milk must be avoided. **5**. Fruits and vegetables liking begins early. Daily variety, diversity in a meal and repeated exposure up to eight times are efficient strategies to increase acceptance of food not well accepted at first. There is no need to add sugar or salt or sugary fluids to the diet. **6**. Respect the child appetite and avoid coercive “clean your plate” feeding practices. Adapt portion of food and not to use food as reward for good behavior. **7**. Limit animal protein intake in early life to reduce the risk of an early adiposity rebound. Growing-up milk should be preferred to cow’s milk in order to limit intake and meet essential fatty acids and iron needs. **8**. The intake of adequate fat containing essential fatty acid should be promoted. **9**. Parents have a model role in feeding, with TV and other screens turned off during meals. **10**. Preventive interventions consisting in promoting physical activity and child has to get sleep sufficiently. In fact, a short sleep duration may be associated with increased risk of developing obesity.

Given the suggestion described in this presentation, concerted public health efforts are needed to achieve the healthy objectives for obesity and nutrition and to fight the childhood obesity epidemic.

### A9 Macronutrient intakes in early life and subsequent risk of obesity

#### Marie Françoise Rolland-Cachera (mf.cachera@uren.smbh.univ-paris13.fr)

##### Paris 13 University, Equipe de Recherche en Epidémiologie Nutritionnelle, Centre de Recherche en Epidémiologie et Statistiques, Inserm (U1153), Inra (U1125), Cnam, COMUE Sorbonne Paris Cité, F-93017 Bobigny, France

There is increasing evidence that early nutrition has an impact on adult health [1]. Several studies have shown an association between high protein intake and later risk of obesity. Besides, there was no evidence of an association between high fat intake in early life and fatness development. Rather, the ELANCE study showed that early low fat intakes were associated with high adult body fat and high serum leptin level at adult age, suggesting a programming of leptin resistance by early fat restriction. An imbalanced diet in early life may program adaptive metabolism through neurodevelopment, metabolic functions or gene expression. These early adaptations may subsequently increase the susceptibility to develop overweight and metabolic diseases when environmental conditions will change. These results have highlighted the inadequate nutrient balance of the infant diet in industrialized countries. Indeed, protein intake represents more than 4 times the protein needs and fat intake is remarkably lower than official recommendations, contrasting with the high fat-low protein content of human milk.

These observations substantiate current recommendations that fat intake should not be restricted in young children. They also underscore the importance of initiating obesity prevention in early life and suggest new directions for investigating the origin of obesity.

**Reference**

1. Rolland-Cachera MF, Scaglioni S. Role of nutrients in promoting adiposity development. In The European Childhood Obesity Group (Eds). The ECOG Free Obesity eBook. Accessed at eu/chapter-nutrition-food-choices-eating-behavior/role-nutrients-promoting-adiposity-development/, 2015

### A10 The burden of childhood obesity in Italy and the results of Nutrintake study

#### Gian Vincenzo Zuccotti (gianvincenzo.zuccotti@unimi.it)

##### Clinica Pediatrica, Ospedale dei Bambini V. Buzzi, Milano, Italy

Childhood obesity is currently one of the most important public health problem. According to World Health Organization, in 2013, more than 42 million of children under the age of five were overweight or obese. In Italian children aged between 8 and 9 years, prevalence of overweight is about 21 %, and prevalence of obesity is nearly 10 %. The Nutrintake 6–36 study has the aim to obtain accurate data about dietary habits in Italian infants and toddlers aged between 6 and 36 months. 390 caucasic children were enrolled in the study. The protein intake resulted to be higher than what is recommended for age. In particular, 50 % of children aged less than 12 months showed a protein intake which is twice higher than what is recommended. 50 % of children aged more than 12 months consumed an amount of protein which was three times higher respect to recommendations. Other results from Nutrintake study were sugars and fats excessive intakes. Practically all infants and children consumed more sugars respect to what is recommended. There was evidence of increasing rate of consumption of saturated fats. In the diet of the majority of children, saturated fats account for more than 10 % of energy. Another important nutritional problem highlighted by the Nutrintake study was the one of excessive sodium intake. Iron deficiency was a nutritional problem identified in almost all the children aged less than 12 months and in the 80 % of those aged more 12 months. The intake of fiber was also inadequate for nearly half of the children aged more than 12 months.

## Growth

### A11 Growth body composition and growth hormone therapy: linear growth

#### Marco Cappa (marco.cappa@opbg.net)

##### Department University Hospital, Unit of Endocrinology an Diabetes, Bambino Gesù Children’s Hospital, IRCCS, Rome, Italy

The concept of height is correlated to a wide variability. The normal height is comprised into a statistically determined range according to the population the subjects are related. The range is comprising between ± 1.9 Standard Deviation from the mean. Because the growth pattern is a complex result of the interaction between genetic and environmental factors, the stature of a child have to be evaluated in relationship with parental height. The most influent environmental factors that may affect growth are wellness, nutritional status and psicoaffective equilibrium. For the above mentioned reason, rarely endocrine disorders are the cause of short stature.

Growth hormone deficiency (GHD) in childhood is characterize by a combination of auxological, clinical, genetic, metabolic and finally hormonal abnormalities. GHD is caused either directly by the complete or relative absence of GH or by secretion of abnormal GH or indirectly by decreased levels of growth factors, which are GH dependent, such as insulin-like growth factor-I (IGF-I).

Recombinant GH therapy is able to restore a normal growth in all patients with GHD, the objective of GH therapy is the normalization of height during childhood, the attainment of normal adult height, and the correction of metabolic abnormalities. The response is related to the age of start therapy, and the GH responsiveness during the first year should be carefully evaluated. GH responsiveness varies largely and is greatly influenced by diagnosis, GH dosing and adherence, adjusting the GH dose to a targeted IGF-I levels may produce a better growth response, improving adherence may also produce a better growth response.

### A12 Early nutrition pattern and late metabolic consequences

#### Manuela Caruso-Nicoletti (manuela.caruso@policlinico.unict.it)

##### Dipartimento di Medicina Clinica e Sperimentale, Sezione di Pediatria, AOU Policlinico-Vittorio Emanuele, Catania, Italy

New scientific insights suggest that nutrition during critical window of time, such as prenatal, early postnatal time and infancy has long-term influences on health beyond childhood. Nutrient supply to the fetus depends on maternal nutritional status and maternal diet. Both maternal undernutrition and overnutrition, as well as maternal dietary imbalance can have detrimental effect on fetal growth and metabolism. Low proteins, excess glucose, low omega-3, micronutrient deficiency (Fe, Zn, Ca, Vit. D, Vit. B12, Folic acid) are associated not only with neonatal outcomes but also with late consequences on insulin sensitivity, energy balance, bone health and neurodevelopment. Long-term metabolism and health are likewise influenced by excessive or deficient intake of some nutrients during infancy, in particular during early postnatal time and the first year of life. Prolonged breast-feeding is the first and best tool to ensure the optimal nutrition during the first months of life; amount and quality of proteins and quality of lipids which are still significantly different in formula vs human milk, have relevant impact on several metabolic function. Introduction of solid foods is associated with a significant reduction and a change in composition of fat intake. Time and modalities of weaning are risk factors for excess protein, reduced fat, in particular high quality fats, reduced/altered micronutrients intake, excess caloric intake. Excess protein intake in infancy influences later weight gain, while excess caloric intake causes early adiposity rebound which is a risk factor for insulin resistance and in turn is associated with risk of developing the metabolic syndrome. Amount and quality of fats in early diet may affect blood lipoproteins (which will influence cardiovascular function) and neurodevelopment. Dairy and whole grains intake in infancy affects bone mineral density in childhood and peak bone mass. Finally, recent studies have suggested that composition of gut microbiota, which seems to play a relevant role in human health, is determined by perinatal factors, including maternal and early postnatal nutrition.

### A13 Nutrition and Insulin-like Growth Factor (IGF) System

#### Elena Inzaghi^1^, Stefano Cianfarani^1,2^

##### ^1^Dipartimento Pediatrico Universitario Ospedaliero Bambino Gesù Children’s Hospital, Università di Roma Tor Vergata, Rome, Italy; ^2^Department of Women’s and Children’s Health, Karolinska Institutet, Stockholm, Sweden

###### **Correspondence:** Stefano Cianfarani (stefano.cianfarani@opbg.net) – Department of Women’s and Children’s Health, Karolinska Institutet, Stockholm, Sweden

**Background:** IGF-I levels have been associated with long-term cardiometabolic risk, whereas IGF-II polymorphisms with BMI, lipid profile and blood pressure.

**Subjects and methods:** 379 obese children (176 F / 203 M, age: 11.2 ± 2.8 years) underwent anthropometric, biochemical and metabolic evaluation. IGF-I serum concentrations were subdivided into ascending tertiles. IGF-II serum levels were assessed in 82 obese and 15 lean patients. Dual X-ray absorptiometry (DXA) was performed in 219 obese patients.

**Results:** IGF-I was directly related with height, pubertal stage, total and truncal lean mass (p < 0.05) and inversely related to waist circumference (WC)/height ratio, BMI, total fat mass, C-reactive protein (CRP) levels (p < 0.05). Subdividing the population according to pubertal status, IGF-I still remained significantly associated with WC and WC/height ratio, after correction for potential confounders. Patients with IGF-I in the lowest tertile had significantly higher WC/height ratio, BMI, total and truncal fat mass percentage, CRP levels and lower lean mass percentage. IGF-II was higher in obese than in lean children and directly related to total cholesterol, LDL-cholesterol, triglyceride levels, and triglyceride/HDL ratio, whereas no association with body composition parameters was found.

**Conclusions:** The associations between IGF-I and IGF-II levels and metabolic and lipid profile suggest that dysregulation of IGF system may be involved in the pathophysiology of cardiometabolic risk in obese children.

### A14 Nutrition of preterm infants

#### Mario De Curtis (mario.decurtis@uniroma1.it)

##### Department of Pediatrics and Pediatric Neuropsychiatry, La Sapienza University of Rome, Italy

Premature infants with a very low birth weight develop a postnatal growth failure in the vast majority of the cases with adverse consequences on both short- and long-term outcomes. Complete enteral feeding is frequently delayed in premature infants and parenteral nutrition represents essential therapeutic option for these infants. Available recommendations suggest starting parenteral nutrition as soon as possible after birth and rapidly attaining adequate intakes with a well-balanced solution, in order to promote anabolism, to improve clinical outcomes, and to avoid biological disorders. A minimum intake of 40–60 kcal/kg/d with 2–3 g/kg/d of amino acids, 1–2 g/kg/d of lipids and sufficient minerals are now recommended from the first hours of life in all premature infants. After immediate postnatal adaptation, intakes of nutrients should rapidly increase during the first week of life. Enteral nutrition should be started since the first 1–2 days of life as minimal enteral feeding (10–30 ml/kg/d) and progressively increased (by 20–30 ml/kg/d) until full enteral feeding is reached (120 kcal/kg/d) and, contemporarily, parenteral nutrition could be stopped. Human milk is the preferred form of enteral nutrition for preterm babies, however fortification with adequate amount of proteins, carbohydrates, lipids, electrolytes and micronutrients should be adopted to respect nutritional needs of these subjects.

### A15 Early nutrition patterns and later metabolic outcomes- Part 1: Genetic and metabolic mechanisms

#### Laura Guazzarotti (guazzarotti.laura@hsacco.it)

##### Department of Pediatric, Luigi Sacco Hospital, University of Milan, Milan, Italy

A growing number of studies focusing on the developmental origin of health and disease have identifed links among early nutrition, epigenetic processes and non-communicable chronic diseases also in later life. Poor nutrition during development could induce changes that would provide short-term advantage but become detrimental under conditions of energy excess. A myriad of exposures in the developmental phases, even within the range of normal birth weight, might induce subtle changes with important implications for later health and disease patterns [1]. The term “predictive adaptive responses” (PARs) has been coined to recognize responses that do not confer an immediate benefit but rather prepare the fetus for the later environment, that is anticipated based on its developmental experience. The mechanistic basis to support this developmental plasticity is provided by the occurrence of epigenetic changes that affect gene expression. Excess or deficits in nutrients, hormones or metabolites may trigger changes in DNA or histone methylation, which in turn suppresses or enhances gene expression; in addition, changes in small noncoding RNA activity act by modulating gene expression [2]. Both maternal under-and over-nutrition may regulate the expression of genes involved in lipid and carbohydrate metabolism. Early postnatal nutrition may also represent a vital determinant of adult health by making an impact on the development and function of gut microbiota and its genetic and metabolic potential [3].

**References**

1. Robinson S, Fall C: **Infant nutrition and later health: A review of current evidence.***Nutrients* 2012,**4**:859–74.

2. Vickers MH: **Early life nutrition, epigenetics and programming of laterlife disease.***Nutrients* 2014,**6**:2165–78.

3. Verduci E, Banderali G, Barberi S, Radaelli G, Lops A, Betti F, Riva E, Giovannini M: **Epigenetic effects of human breast milk.***Nutrients* 2014,**6**:1711–24.

### A16 Diagnosis of metabolic disease by imaging techniques

#### Lorenzo Iughetti (lorenzo.iughetti@unimore.it)

##### Postgraduate School of Pediatrics, University of Modena and Reggio Emilia, Modena, Italy

An increasing number of studies focus on fat distribution and its associations with metabolic risk, in interaction with genetics, environment and ethnicity, in children. The link between obesity and metabolic disease risk is driven by body fat distribution and ectopic fat deposition, but the only way to accurately visualize and quantify specific fat depots is state-of-the-art imaging techniques, like computed tomography (CT) and magnetic resonance imaging (MRI). Recent advances in imaging techniques, especially in MRI, have made it possible to measure specific fat depots such as visceral abdominal fat (visceral adipose tissue [VAT]), subcutaneous abdominal fat (subcutaneous adipose tissue [SAT]) and ectopic fat depots including hepatic fat fraction (HFF), pancreatic fat fraction (PFF) and intramyocellular fat (IMCL fat). Abdominal as well as ectopic fat depots are present already in childhood and contribute to abnormal metabolic parameters, starting early in life. Visceral, hepatic and intramuscular fat seem to be interrelated but their patterns as well as their independent contribution on metabolic risk are not clear. Girls tend to accumulate more TBF and SAT during and after puberty, depositing fat preferentially in the gynoid and extremity regions. In contrast, pubertal and postpubertal boys tend to deposit more fat in the abdominal region, particularly in the VAT depot. Sexual maturation significantly influences TBF, VAT and SAT. Some ethnic-specific characteristics are also prevalent. Further researches are needed in childhood obesity by using imaging techniques such as magnetic resonance imaging and computed tomography. These imaging methods can provide a better understanding of fat distribution and its relationships with metabolic risk, compared to less detailed fat and obesity assessment. However, studies on bigger samples and with a prospective character are warranted.

### A17 Nutrition, growth and cardiovascular diseases

#### Francesco Chiarelli^1,2^, Laura Comegna^1^, Simone Franchini^1^

##### ^1^Department of Pediatrics, University of Chieti, Chieti, Italy; ^2^Clinical Research Centre, University of Chieti, Chieti, Italy

###### **Correspondence:** Francesco Chiarelli (chiarelli@unich.it) – Clinical Research Centre, University of Chieti, Chieti, Italy

Childhood obesity has reached epidemic proportions around the world both in western and developing countries [1]. Several factors modulate the risk of obesity during life, such as demographic factors, maternal behaviors (diet, smoking), birth weight and lactation choices, nutrition and physical exercise. Obesity genes should be also considered [2].

Metabolic impairment seems to track from childhood into adulthood and onward, so risk factors already altered in youth will persist and enhance risks for both cardiovascular events and diabetes in adulthood. This concept has been demonstrated for body mass index (BMI) and the risk of hypertension and dyslipidemia later in life [3, 4]. Prenatal, perinatal and neonatal life may predispose to adult health outcomes and cardiovascular risk. Maternal under- or over-nutrition, dietary imbalance, BMI and weight gain during pregnancy cause systemic insulin resistance (IR) in the offspring through several molecular mechanisms, easing the onset of metabolic syndrome (MS) or type 2 diabetes (T2D) [5]. These habits influence body size and metabolic pathways in fetus and infants: children born small-for-gestational-age or after an intrauterine growth restriction seem to have a higher risk of T2D and MS during adulthood compared to appropriate-for-gestational-age infants.

Nutrition in infancy influences the cardio-metabolic profile by cardiovascular developmental adaptations in response to specific infant feeding patterns. Breastfeeding is related to a lower weight-SDS in pediatrics but also in adulthood when compared with bottle-fed infants [6]. Moreover, never-breastfed children present a worse cardiovascular profile, with smaller cardiac mass and diameter of heart chambers. Duration and exclusivity of breastfeeding do not influence these characteristics. On the other hand, age at introduction of solid foods seems to be negatively associated with systolic and diastolic blood pressure [7].

The adiposity rebound is a physiologic increase in BMI during childhood after a physiologic nadir in infancy. The earliest the onset of adiposity rebound, the highest the risk of later obesity and lipoprotein profile representative of IR. This phenotype consists of elevated triglycerides, apolipoprotein B, atherogenic index and reduced HDL-cholesterol [8]. High energy intake and high consumption of sweetened drinks in childhood are prospectively associated with raised obesity risk during childhood [9, 10]. Failure to attain fat balance may contribute to obesity development even without excessive energy intake. The highest the percentage of daily energy derived from fat intake, the most represented visceral, truncal and abdominal adiposity. A great consumption of fruit and vegetables reduces risk of all cause mortality, particularly cardiovascular mortality [11, 12].

**References**

1. Han JC, Lawlor DA, Kimm SY: **Childhood obesity.***Lancet* 2010, **375**:1737–48.

2. Poston L: **Maternal obesity, gestational weight gain and diet as determinants of offspring long term health.***Best Pract Res Clin Endocrinol Metab* 2012, **26**:627–39.

3. Tirosh A, Shai I, Afek A, Dubnov-Raz G, Ayalon N, Gordon B, Derazne E, Tzur D, Shamis A, Vinker S, Rudich A: **Adolescent BMI trajectory and risk of diabetes versus coronary disease.***N Engl J Med* 2011, **364**:1315–25.

4. Jounala M, Magnussen CG, Berenson GS, Venn A, Burns TL, Sabin MA, Srinivasan SR, Daniels SR, Davis PH, Chen W, Sun C, Cheung M, Viikari JS, Dwyer T, Raitakari OT: **Childhood adiposity, adult adiposity, and cardiovascular risk factors.***N Engl J Med* 2011, **365**:1876–85.

5. Duque-Guimaraes D, Ozanne SE: **Nutritional programming of insulin resistance: causes and consequences.***Trends Endocrinol Metab* 2013, **24**:525–35.

6. Ong KK, Preece MA, Emmett PM, Ahmed ML, Dunger DB; ALSPAC Study Team: **Size at birth and early childhood growth in relation to maternal smoking, parity and infant breast-feeding: longitudinal birth cohort study and analysis.***Pediatr Res* 2002, **52**:863–7.

7. de Jonge LL, Langhout MA, Taal HR, Franco OH, Raat H, Hofman A, van Osch-Gevers L, Jaddoe VW: **Infant feeding patterns are associated with cardiovascular structures and function in childhood.***J Nutr* 2013, **143**:1959–65.

8. Koyama S, Ichikawa G, Kojima M, Shimura N, Sairenchi T, Arisaka O: **Adiposity rebound and the development of metabolic syndrome.***Pediatrics* 2014, **133**:e114-9.

9. Levy-Marchal C, Arslanian S, Cutfield W, Sinaiko A, Druet C, Marcovecchio ML, Chiarelli F; ESPE-LWPES-ISPAD-APPES-APEG-SLEP-JSPE; Insulin Resistance in Children Consensus Conference Group: **Insulin resistance in children: consensus, perspective, and future directions.***J Endocrinol Metab* 2010, **95**:5189–98.

10. de Ruyter JC, Olthof MR, Seidell JC, Katan MB: **A trial of sugar-free or sugar-sweetened bevereges and body weight in children.***N Engl J Med* 2012, **367**:1397–406.

11. Labayen I, Ruiz JR, Ortega FB, Huybrechts I, Rodriguez G, Jimenez-Pavon D, Roccaldo R, Nova E, Widhalm K, Kafatos A, Molnar D, Androutsos O, Moreno LA: **High fat diets are associated with higher abdominal adiposity regardless of physical activity in adolescents; the HELENA study.***Clin Nutr* 2014, **33**:859–66.

12. Wang Y, Ouyang Y, Liu J, Zhu M, Zhao G, Bao W, Hu FB: **Fruit and vegetable consumption and mortality from all causes, cardiovascular disease, and cancer: systematic review and dose–response meta-analysis of prospective cohort studies.***Br Med J* 2014, **349**:g4490.

### A18 Body fat mass and gender

#### Laura Perrone, Giuseppina Rosaria Umano

##### Department of Women and Children and General and Specialized Surgery, Seconda Università degli Studi di Napoli, Naples, 80138, Italy

###### **Correspondence:** Laura Perrone (laura.perrone@unina2.it) – Department of Women and Children and General and Specialized Surgery, Seconda Università degli Studi di Napoli, Naples, 80138, Italy

Human adipose tissue is classically characterized as white and brown and beige. The vast majority of human adipose tissue is of the white type; this type of fat is mainly located beneath the skin (subcutaneous adipose tissue) but also around internal organs (visceral adipose tissue, VAT) [1].

The radiological classification of VAT is performed according to the body region where fat is deposited and it includes: intrathoracic (ITAT) and intraabdominal (IAAT) adipose tissue. IAAT can be distinguished further into intraperitoneal and extraperitoneal adipose tissue. ITAT is classified into epicardial adipose tissue, which is deposited beneath the visceral pericardium, and extrapericardial, which is deposited outside the parietal pericardium.

Adipose tissue disorders (e.g.: obesity and lipodystrophy) cause alterations to adipose tissue distribution and function. The resulting changes appear to induce profound consequences for basal systemic inflammation and insulin sensitivity [2].

Recently, a concept has successively developed that a significant number of adult humans possess active brown adipose tissue (BAT). In the newborn, BAT is essential for ensuring effective adaptation to the extrauterine environment. BAT possesses a unique uncoupling protein, UCP1, which is responsible for the generation of large amounts of heat at birth. Then, some depots are replaced by white fat [3].

It is established that BAT is primarily located within the supraclavicular regions in adults of all ages. Maximal heat production by BAT is of the order of 300 W/kg compared with 1 W/kg in all other tissues. Recent studies have demonstrated the feasibility of using infrared thermography as a safe, reproducible, and robust technique for measuring the temperature of the skin overlying BAT depots. This technique has demonstrated that BAT function is greater in young children than adolescents and adults [4], with a negative relationship between BMI and BAT activity in children [5].

The last few years have seen great advances in our understanding of inducible thermogenic adipose tissue, also referred to as beige fat. These cells can robustly respond to many stimuli (including cold and irisin) to activate thermogenesys to levels similar to those seen in BAT, resulting in increased energy dissipation. When energy intake exceeds energy expenditure, the surplus energy can be stored in the form of lipid, and beige fat cells take on a more “white” morphology [6].

This growing recognition of nonadrenergic signals to induce BAT activity may be of most interest to the development of pharmacological strategies to promote healthy metabolic consequences of BAT activation [7].

**References**

1. Alexopoulos N, Katritsis D, Raggi P: **Visceral adipose tissue as a source of inflammation and promoter of atherosclerosis.***Atherosclerosis* 2014,**233**:104–12.

2. Makki K, Froguel P, Wolowczuk I: **Adipose tissue in obesity-related inflammation and insulin resistance: cells, cytokines, and chemokines.***ISRN Inflamm* 2013:139239.

3. Symonds ME, Pope M, Sharkey D, Budge H: **Adipose tissue and fetal programming.***Diabetologia* 2012,**55**:1597–606.

4. Symonds ME, Henderson K, Elvidge L, Bosman C, Sharkey D, Perkins AC, Budge H: **Thermal imaging to assess age-related changes of skin temperature within the supraclavicular region co-locating with brown adipose tissue in healthy children.***J Pediatr* 2012,**161**:892–8.

5. Robinson L, Ojha S, Symonds ME, Budge H: **Body mass index as a determinant of brown adipose tissue function in healthy children.***J Pediatr* 2014,**164**:318–22.

6. Wu J, Cohen P, Spiegelman BM: **Adaptive thermogenesis in adipocytes: is beige the new brown?***Genes Dev* 2013,**27**:234–50.

7. Villarroya F: **Irisin, turning up the heat.***Cell Metab* 2012,**15**:277–8.

### A19 Lifestyle interventions for an appropriate birth weight

#### Elisabetta Petrella, Raffaele Bruno, Valentina Bertarini, Giulia Pedrielli, Isabella Neri, Fabio Facchinetti

##### Mother-Infant Department, University of Modena and Reggio Emilia, Italy

###### **Correspondence:** Elisabetta Petrella (petrella.elisabetta@gmail.com) – Mother-Infant Department, University of Modena and Reggio Emilia, Italy

**Background.** A high pre-pregnancy body mass index (BMI) and/or an excessive gestational weight gain (GWG) are associated with many unfavorable maternal-neonatal outcomes, in particular with gestational diabetes mellitus (GDM) and excessive birth weight [1].

A recent systematic review reported no effect of lifestyle interventions among overweight/obese women on the rate of GDM and large for gestational age (LGA); however, included studies were too heterogeneous. [2] The specific type of intervention seems of the utmost importance and an important confounding factor evaluating the effect of behavioral changes is the adherence to the suggested prescriptions.

**Materials and methods.** Women with BMI ≥ 25 (n = 191) were randomized between 9^th^-12^th^ week to *Control group* (C = 95: simple nutritional booklet about lifestyle) or *Experimental group* (EXP = 96: detailed low-GI diet prescribed by dietitian with an intake of 1700 Kcal/day plus moderate physical activity). Weight at enrollment and at each follow-up visit (planned at 16^th^, 20^th^, 28^th^ and 36^th^ week) was measured. At baseline and at the 36^th^ week women filled-in a Food Frequency Questionnaire (FFQ).

**Results.** Socio-demographic characteristics at randomization and BMI categories were equally distributed. Miscarriage occurred in 13 pregnancies (6.8 %) while 47 women dropped out after randomization, leaving 131 women (C group: 62, EXP group: 69). Comparing the FFQ, we observed in group EXP significant positive changes in the consumption of every investigated food, while in group C consumption changed only for few of them. More women in group EXP were compliant to a proper nutrition, as prescribed. Results for the main outcomes are summarized in Table 1. No difference was found regarding GWG; however the rate of GDM, LGA were lower in group EXP, while the rate of SGA was unaffected by the intervention.

**Conclusions.** Prescription of a low-glycemic diet with caloric restriction is effective in modifying nutritional habits, improving the adherence to the behavioral program and reducing the incidence of GDM and LGA babies among overweight/obese women.

**Trial registration**

Clinicaltrials.gov NCT01783210.

**References**

1. Yu Z, Han S, Zhu J, Sun X, Ji C, Guo X: **Pre-pregnancy body mass index in relation to infant birth weight and offspring overweight/obesity: a systematic review and meta-analysis.***PLoS One* 2013, **8**:e61627.

2. Oteng-Ntim E, Varma R, Croker H, Poston L, Doyle P: **Lifestyle interventions for overweight and obese pregnant women to improve pregnancy outcome: systematic review and meta-analysis**. *BMC Med* 2012,**10**: 47.

**Table 1 (abstract A19).** Maternal and neonatal outcomes

	Group EXP (69)	Group C (62)	*p*-value
GDM	13 (18.8 %)	23 (37.1 %)	0.019
Pregnancy induced hypertension	2 (2.9 %)	13 (21 %)	0.001
Birthweight (grams)	3432.5 ± 333.7	3512.3 ± 447.3	0.246
LGA (≥90° percentile)	1 (1.4 %)	7 (11.3 %)	0.019
Macrosomic newborns (≥4000 g)	2 (2.9 %)	7 (11.3 %)	0.058
SGA (≤10° percentile)	6 (8.7 %)	5 (8.1 %)	0.897

### A20 Nutrition, growth and body composition

#### Flavia Prodam (flavia.prodam@med.uniupo.it)

##### Division of Pediatrics, Department of Health Sciences, Università del Piemonte Orientale, Novara, 28100, Italy; Endocrinology, Department of Translational Medicine, Università del Piemonte Orientale, Novara, 28100, Italy

Longitudinal growth from prenatal period to adulthood is a complex phenomenon. Initiation, rate, and cessation of growth are widely orchestrated by many factors, in particular hormones and nutrients. Most studied hormones included growth hormone (GH) and insulin-like growth factor I (IGF-I), ghrelin, insulin, leptin, thyroid hormones, glucocorticoids and vitamin D, while among micronutrients zinc, and iron. It is clear that malnutrition impairs linear growth as demonstrated in many studies in developing countries as well as that refeeding is usually followed by a catch-up growth. Moreover, it is well known that fasting and feeding acutely ad chronically modulate the secretion of several hormones. Weight gain and subsequent linear growth are associated with an increase in IGF-1 in children with malnutrition or amelioration of chronic diseases. Although specific ghrelin activities in the growth plate have not yet been identified, ghrelin secretion is refractory to the inhibition by feeding in lean infants and children, suggesting a specific anabolic role of the “hunger hormone” in this phase of life. Extreme insulin resistance seems associated with growth retardation maybe due to decreased energy supply or lack of specific actions of insulin on the growth plate. Prolonged vitamin D deficiency resulting in rickets is seen mainly during rapid growth. However, in healthy children, there is no consistent association of 25OH vitamin D concentrations with growth and skeletal development, suggesting a role of more factors in the relationship between vitamin D and bone. Zinc deficiency which decreases circulating IGF-1 concentration, and iron deficiency both result in growth retardation. Recent studies suggest that markers of bone and collagen formation may be useful nutritional biomarkers for growth outcomes.

Despite this evidence, the exact mechanisms by which the body signals the long bones to grow is still unclear, although several mediators are under investigation. MicroRNAs, transcription factors, energy-sensing enzymes (mTOR), autophagy and epigenetic mechanisms are studied as links between nutrients and hormones in the modulation of linear growth and of nutrient-induced catch-up growth. On the other hand, clear data in human studies are still lacking, in particular in children living in countries where the environment is not characterized by a condition of food restriction. Further efforts are needed to understand the interplay between nutrition and growth.

### A21 Nation-specific reference growth charts in the daily practice

#### Alessandro Sartorio, John M.H. Buckler, Nicoletta Marazzi

##### Istituto Auxologico Italiano, Milan, Italy; Istituto Auxologico Italiano, Verbania, Italy

###### **Correspondence:** Alessandro Sartorio (sartorio@auxologico.it) – Istituto Auxologico Italiano, Milan, Italy

The growing phenomenon of immigration, which affects all industrialized countries, more and more frequently exposes the paediatricians/auxologists to the problem of assessing growth and development of children and adolescents of different ethnicities in their daily practice. To interpret correctly the anthropometric data on the growth charts, it is fundamental that these are reliable and appropriate for that particular subject or population. Only with the use of specific growth charts, which allow a comparison with the reference population, it is possible to understand whether the growth of a child is within the normal limits or it requires targeted diagnostic controls and, if necessary, proper treatments. Unfortunately not all the countries, especially the poorest ones, have their own growth charts, so it is virtually impossible to have the "ideal" growth charts for all the nations of the world. A growth chart which is representative for a given nation can be used (with obvious limitations) also for the evaluation of children of the neighboring countries, provided they have comparable socio-economic characteristics.

"Growing in the world: a collection of growth charts" is a project started in 2002, periodically updated thanks to the ongoing relationships between our Institute and the embassies, consulates, hospitals, universities, scientific societies and international organizations. The updated 2015 edition, distributed free of charge to the Italian hospital and university centers that deal with growth and physical development, contains 502 growth charts of height and weight from 83 different countries worldwide, with a coverage of over 80 % of world population (for some continents up to 90 %).

Nation-specific growth charts of a country represent the reference for that specific population, obviously not being an international "gold standard". For this reason, WHO has developed new standards that show how children should grow in all countries of the world in best life conditions (optimal nutritional conditions, absence of external constraints, healthy environment). According to WHO, the sample collected in the six countries participating in the study (Brazil, Ghana, India, Norway, Oman, USA) has allowed us to build an international standard "which all growing children should aim at." The WHO concept that children of every race and background have the same growth potential if placed in the ideal conditions was received coldly by many paediatricians/auxologists, which signed a shared document [1] in favour of the nation-specific growth reference charts - based on the fact that children and adolescents growth is grossly different in different countries and different ethnic groups even if the same socio-economic conditions are present, as well as on the view that diversity is an asset to be safeguarded and not a defect to be eliminated. Obviously, the use of nation-specific reference charts or of a single international standard in the evaluation of growth and development of a child entails significant differences in the evaluation of weight, BMI and height centiles (with consequences also on possible substitutive treatments) and also ripples on the proper assessment of the weight excess and obesity prevalence in the populations. If the phenomenon of immigration of children continues in the next decades (and/or it intensifies further), we will be requested to build new reference charts for height, weight and BMI at least for the main immigrant ethnic groups living in our countries. The influence of new lifestyles in the new host countries can obviously affect (as time goes by) the typical growth pattern of the countries their families are from.

References

1. Milani S, Buckler JMH, Kelnar CJH, Benso L, Gilli G, Nicoletti I, Faglia G, Radetti G, Bona G, Schonbeck Y, Van Buuren S, Hermanussen M, Grugni G, Marazzi N, Júlíusson PB, Roelants M, Hoppenbrouwers K, Hauspie R, Bjerknes R, Lejarraga H, Sartorio A: **The use of local reference growth charts for clinical use or a universal standard: a balanced appraisal.***J Endocrinol Invest* 2012, **35**:224–226.

### A22 Growth patterns in inflammatory bowel diseases (IBD) and in cystic fibrosis (CF)

#### Maria E. Street, Viviana D. Patianna, Paola Accorsi, Sara Lo Scocco, Sergio Amarri

##### Department of Paediatrics, IRCCS- Arcispedale S. Maria Nuova, Reggio Emilia, Italy

###### **Correspondence:** Maria E. Street (mariaelisabeth.street@asmn.re.it) – Department of Paediatrics, IRCCS- Arcispedale S. Maria Nuova, Reggio Emilia, Italy

Impaired growth and delayed pubertal development are often described in chronic inflammatory diseases. Growth delay can be mainly attributed to calorie and protein malnutrition due to anorexia, malabsorption and increased resting energy expenditure [1].

With respect to IBD, the incidence of ulcerative colitis is considered to vary from 10-20/10^5^ cases per year for Northern America and Northern Europe. For Crohn's disease the respective value is 5-10/10^5^ for the same geographical area [2]. In these children, body composition seems to give a better indication of nutritional status than measures of anthropometry. In recent years treatment has greatly improved thanks to the use of biologic therapy that targets specific mediators of the pro-inflammatory process with effects on growth and skeletal development. In IBD mainly two anti-TNF alfa monoclonal antibodies are used, Infliximab and Adalimumab. An increase in growth velocity has been reported in good responders to Infliximab treatment as a result of disease control, with better results observed in those who had never been on glucocorticoids. Adalimumab has been shown also to be effective in those children who enter remission. Biologic therapy seems to have mostly an effect on bone formation, and would increase both fat mass and fat free mass [3]. In IBD, the aetiology of pubertal delay is multifactorial and hypogonadism may encompass a range of abnormalities of the hypothalamic/pituitary/gonadal/end-organ axis as hypogonadotrophic hypogonadism, abnormality of sex steroid synthesis or an abnormality of sex steroid action [4, 5].

Growth delay is often a feature of patients with CF. Poor growth in CF has been largely ascribed to malnutrition/malabsorption, deteriorating pulmonary function and reduced insulin secretion [6, 7]. Stature has been regarded as a prognostic factor in CF survival [8]. Improved nutritional conditions and a better treatment of this condition have led over time to an increase in length, and in weight for length in small children. In elder subjects, the BMI percentile has clearly increased over years to values similar to the average of the population of referral, and the percentage of patients underweight has halved since 1998 [6]. Specific changes have been described related with the inflammatory status. Low IGF-I serum concentrations have been repeatedly reported. We have shown a relationship between inflammatory status and the IGF system, and an effect of these interactions on longitudinal growth, in detail with the change in height SDS over one year of observation [9]. Moreover, a role for insulin in growth was identified which led us to conclude that better control of inflammation and preservation of insulin secretion would benefit growth in these patients.

**References**

1. Hill JR: **Update on nutritional status, body composition and growth in paediatric inflammatory bowel disease**. *World J Gastroenterol* 2014,*20*: 3191–3197.

2. Loftus EV: **Clinical epidemiology of inflammatory bowel disease: Incidence, prevalence, and environmental influences.***Gastroenterology* 2004, **126**: 1504–1517.

3. Malik S, Ahmed SF: **Biologic therapy and its effect on skeletal development in children with chronic inflammation.***Expert Rev Endocrinol Metab* 2010,**5**: 733–740.

4. Mason A.Malik S. Russell RK, Bishop J, McGrogan P, Ahmed SF: **impact of inflammatory bowel disease on pubertal growth.***Horm Res Paediatr* 2011, **76**:293–299.

5. Mason A., Malik S., McMillan M, McNeilly JD, Bishop J, McGrogan P, Russell RK, Ahmed FS: **A prospective longitudinal study of growth and pubertal progress in adolescents with inflammatory bowel disease.***Horm Res Paediatr* 2015, **83**: 45–54.

6. Cystic Fibrosis Foundation.Patient Registry Annual Data Report *2013*. Cystic Fibrosis Foundation, Bethesda, MD. Available online at: www.cff.org/research/ClinicalResearch/PatientRegistryReport (accessed 26 May 2015).

7. Littlewood JM, Wolfe SP: **Control of malabsorption in cystic fibrosis**. *Paediatric Drugs* 2000, **2**: 205–222.

8. Beker LT, Russek-Cohen E, Fink RJ: **Stature as a prognostic factor in cystic fibrosis survival**. *J Am Diab Assoc* 2001, **101**: 438–442.

9. Street ME, Spaggiari C, Volta C, Ziveri MA, Viani I, Rossi M, Pisi G, Grzincich GL, Bernasconi S: **The IGF system and cytokine interactions and relationships with longitudinal growth in prepubertal patients with cystic fibrosis.***Clin Endocrinol* 2009, **70**: 593–598.

## Physical activity

### A23 Newborn in the digital era and their body feeling: physical exercise to counteract hyperphagia

#### Alberto Pellai

##### Medical Sciences Department, University of Milano, Milano, Italy

As adults, we are growing up the first generation of newborns in the digital area. As behavioral epidemiology confirms, their technological habits are so intense to bring them to pursue a constantly increasing high-tech lifestyle. Every day, they stay online for many hours and this has an impact on their well being, influencing all the three dimensions of health: biological, psychological and social. This changes have led to a new form of eating disorder defined “conditioned hypereating” (D. Kessler). Why do children eat beyond their needs? Why have they lost the physiological sensations for appetite and fullness? Is physical activity and sport participation a way to counteract the risks of “conditioned hypereating”? The presentation will describe the neuroscience’s evidences explaining how “conditioned hypereating” can be developed and prevented among children. Besides, it will be discussed how to help digital natives to keep in touch with their body feelings and sensations as a way to decrease the obesity epidemics.

Considering that “managing nutrition and weight today requires skillpower, not willpower” (D. Katz), the concept of skillpower will be analyzed and discussed so to define what preventions strategies are needed and effective to prevent conditioned hypereating.

### A24 Nutrition, young athletes and effects of exercise. Practical suggestions

#### Giampiero Merati^1,2^

##### ^1^Department of Biomedical Sciences for Health, Università degli Studi di Milano, Milan, Italy; ^2^Sport Medicine Center, Fondazione Don Gnocchi, Milan, Italy

Sports nutrition in childhood must satisfy the needs of training together, and not at the expense, to those of growth. Football is the most popular sport in the world among children and adolescents, giving benefits to bone, muscle, cardiovascular and metabolic systems. Furthermore, soccer training prevents the formation of new fat cells, reducing overweight and obesity.

Energy expenditure in football is about 4 and 9 calories/minute for the children, and is influenced by weight, body composition, sex, role (a midfielder spends more energy than a central defender, who in turn spends more of a goalkeeper) and game category. Heart rate may be a good surrogate of energy consumption in children. Vitamin D, calcium, iron, vitamins A, C and the B group, magnesium, fibres and carbohydrates are extremely important in sports nutrition for children: the diet might be high in carbohydrates (6–10 g per kg, at least 50-55 % of total calories), which are essential also in post-game recovery. As regards proteins, many studies in Europe have shown that the daily consumption of proteins in children is higher than recommended. Lipids should not exceed 30 % of total calories. Finally, hydration is critical, as children are less efficient than adults in removing heat form the body. As regards supplements, the most used are multivitamins and creatine. However, a varied diet provides all the nutrients needed by sport activity in the age of development.

### A25 Physical exercise as a way to prevent criminality in minors and teenagers

#### Isabella Merzagora

##### Medicina Legale, Dipartimento di Scienze Biomediche per la Salute, University of Milan, Milan, Italy

Besides physical and mental health, it also exists a social health, and it’s proven that physical exercise has an important role in it as well, namely in preventing minors and teenagers criminality.

The study verified that exercise impinges on criminological factors, strenghtening self-esteem and identity, decreasing sense of inferiority and inadequacy, curbing impulsiveness and opposition, meeting the need to socialize, teaching cooperation and fair-play.

It also has been noted the positive effect physical exercise has on restricting victimisation, particularly bullying, mainly for children (5–7 years old).

However, criminological studies found out some differences: not all the physical activities have the same effects, and not always the effect is a positive one.

Conclusions are that physical exercise is one of the tool able to prevent both behaviour disorders in children and teenagers and outright criminality, contributing to social health, with percentages that go from 6.7 % to 60 % of decreasing criminality rates and number of minors arrested.

Finally, Authors point out that the delinquency prevention projects based on involving young people in physical activities have much lower costs than the expenses caused by all the different forms of criminality (health care expenses; loss done to robberies and damages; psychological costs in terms of personal sufferings for the victims involved; etc.).

### A26 The measurement of daily energy expenditure in children. Evaluation of a new wrist portable device vs breath-by-breath metabolimeter

#### Susanna Rampichini^1^, Arsenio Veicsteinas^2^

##### ^1^Department of Biomedical Sciences for Health, University of Milan, Italy; ^2^Physiology, University of Milan, Italy

###### **Correspondence:** Susanna Rampichini (susanna.rampichini@unimi.it) – Department of Biomedical Sciences for Health, University of Milan, Italy; Arsenio Veicsteinas (arsenio.veicsteinas@unimi.it) – Department of Physiology, University of Milan, Italy

Inactivity is one of the major factors associated to most of the cardiovascular and metabolic diseases. Many new wearable devices are becoming available to monitor physical activity of people with high risk factors of disease. Aim of the study was to evaluate a new multi-sensors monitor for energy expenditure (EE) assessment in overweight children during light to moderate activities. Nineteen overweight children (10 males; age: 12.8 ± 1.8 yrs; body mass: 75.3 ± 12.7 kg; body mass index: 28.6 ± 2.6 kg/m^2^; mean ± SD) were enrolled. The subjects were tested during: i) walking at comfortable speed (SS) and ii) walking at faster speed (FS). EE during the entire tasks was assessed by both indirect calorimetry (IC) measuring the oxygen consumption through a metabolimeter on a breath-by-breath basis, and by a newly developed wrist monitor (WM) measuring skin conductance, heart rate, surface temperature and acceleration. EE measured by IC during SS and FS was 4.08 ± 0.69 and 5.36 ± 0.92 METs, respectively. A two-ways ANOVA for repeated measures showed no differences in EE assessed by IC and WM and an expected significant increment of the values measured during FS compared to SS (p < 0.001). The correlation coefficient between EE obtained by the two methods was 0.61. We conclude that wrist portable device is a very useful tool for indirect EE monitoring in children and that strongly correlates with oxygen consumption.

## Probiotics

### A27 Probiotic and inflammasomes

#### Mario Clerici^1,2^

##### ^1^School in Molecular and Translational Medicine, University of Milano, Italy; ^2^Department of Molecular medicine and Imaging for Rehabilitation, IRCCS SM Nascente, Fondazione Don C Gnocchi, Milano, Italy

**Background:** Immune activation contributes to the persistent inflammation associated with metabolic dysfunction in obesity. There is, however, little information on inflammatory and immune gene regulation in obesity.

**Methods:** 22 obese children and adolescents and 18 age-matched normal weight controls were analyzed. Obese subjects underwent an 18-months protocol based on intensive lifestyle modification (dietary regimen, physical activity and behavioral interventions). Expression of inflammasome genes, plasma concentration of pro-inflammatory cytokines (IL-1ß, IL-18), and indexes of microbial translocation (LPS, sCD14, and I-FABP) were studied at baseline in all subjects, and 18 months later in obese individuals.

**Results:** LPS-stimulated expression of inflammasome, Nod-like receptors, and proinflammatory cytokines genes, as well as IL-1ß, IL-18, LPS, and sCD14 plasma con- centration were significantly increased in obese individuals at baseline. Intensive lifestyle modification resulted in a normalization of parameters in individuals in whom significant weight loss was recorded after 18 months.

**Conclusions:** Obesity is characterized in children and adolescents by the activation of the inflammasome and by an alteration of gut permeability. Successful dietary intervention dampens inflammation suggesting that inhibition of the inflammasome may be a potential therapeutic strategy in obesity.

### A28 Probiotics and newborns

#### Paolo Manzoni, Elena Tavella, Elena Boano, Daniele Farina

##### Neonatology and NICU, S. Anna Hospital, Torino, Italy

Preterm neonates in NICU have a high risk for intestinal disorders with proliferation of pathogenic microflora. Treatment with antibiotics, total parenteral nutrition, or nursing in incubators may in fact delay or impair the physiological gut colonization process. Preterms thus acquire commensals such as bifidobacteria more slowly and may acquire gut pathogenic colonization from the NICU.

For all these reasons, the digestive tract is regarded as the most important reservoir and site for colonisation by all kinds of pathogens and subsequent sepsis in preterms.

Probiotics can restore normality of gut microbiota, and prevent its disturbances in humans including newborns.

Studies in mice have shown that selected probiotic strains reduce both enteric colonization and systemic infections by E. Coli and fungi. Such strains may act at several levels simultaneously: exclusion of pathogens by competition, prevention of adhesion, reduction of their ability to colonise the mucosa through enhanced IgA responses, changes in mucosal permeability increasing the barrier effect, and immunomodulation with modification of local immune responses and of host response to fungal and bacterial toxins and products.

To date, only a few clinical trials have reported the outcomes of preterm neonates given probiotics: these studies consistently show beneficial effects of some probiotic mixtures in promoting gastrointestinal maturity and function, preventing fungal colonization, improving feeding tolerance, and reducing the incidence of necrotizing enterocolitis.

In the light of this evidence, probiotic administration to preterm infants in NICU is an area of current interest and updates. However, concerns exist about safety and tolerability of probiotics administered to preterm infants, as long-lasting administration of living microrganisms to immature patients might translate into breakthrough infections by the same organisms. This will be a major area of research in the future.

### A29 Relationship between gut microbiota and obesity

#### Fabio Pace

##### UOC Gastroenterologia Ospedale “Bolognini”, Seriate, BG, Italy

The worldwide obesity epidemic is stimulating efforts to identify host and environmental factors that affect energy balance. One possible contributing factor to the pathophysiology of obesity might be the gut microbiota. Studies conducted in experiment animal and also in humans, comparing the distal gut microbiota of obese individuals with those of lean human volunteers, have revealed that obesity is associated with changes in the relative abundance of the two dominant bacterial divisions, the Bacteroidetes (B) and the Firmicutes (F). Such changes directly affect the host metabolism by an increased capacity to harvest energy from the diet, with multiple mechanisms, involving an increased absorption of monosaccharides, an increased deposition of lipid in adipocytes, or the induction of pro-inflammatory cytokines, which induces a desensitization of insulin receptor signaling. However, it is still controversial whether alterations in the microbiota are a cause or consequence of obesity, since diet is a potent modulator of the composition of gut microbiota, as shown by comparative analyses of the fecal microbiota of rural African children with that of European children, which showed in the former a significant enrichment in B and depletion in F. Whether cause or consequence of diet, the “obese microbiota” is a transmissible trait. Thus, the gut microbiota is a contributing factor to the pathophysiology of obesity.

